# Automatic pain assessment from facial action units in ICU patients via various machine learning models

**DOI:** 10.1038/s41598-026-57148-3

**Published:** 2026-06-13

**Authors:** Zhen Cui, Xin Yuan, Hao Wang, Ying Bai, Liu Zhang, Fan Zhang, Bo Ouyang

**Affiliations:** 1 Beijing Jishuitan Hospital, Capital Medical University, Department of Critical Care Medicine, Beijing, 100035 China; 2 Hefei University of Technology, School of Management, Hefei, 230009 China

**Keywords:** FACS, ICU, Action unit combinations, Automated pain detection, Machine learning, Diagnosis, Biomarkers, Diagnostic markers

## Abstract

Pain represents a critical vital sign monitored in intensive care unit (ICU) patients. The facial action coding system (FACS) defines facial action units (AUs) and provides a structured framework for pain recognition. Currently, the most broadly used pain assessment method is the pain intensity scale developed by Prkachin and Solomon (PSPI), which relies on predefined AUs to quantify facial expressions. However, due to the influence of underlying diseases and facial texture variations in ICU patients, AUs can fail to transfer to clinical settings accurately. To address this problem, this study uses video sequences of pain states collected from 61 ICU patients under resting, daily, and procedural conditions by using an advanced AU detection system. By evaluating the AUs with statistical features and various classification models, this study identifies six key AUs that outperform the PSPI’s predefined AUs in terms of accuracy, precision, recall, and F1-score metrics. Further, this study explores the performance of various temporal self-learning networks in the pain assessment task, thus further validating the effectiveness of the identified AU combination. The results presented in this study demonstrate that using AU dynamic learning in combination with deep temporal analysis can improve the reliability of clinical pain assessment. Finally, this study offers a promising approach for automated pain monitoring systems in ICU settings.

## Introduction

Pain is one of the primary symptoms assessed in intensive care unit (ICU) patients, among whom 70% experience moderate to severe pain^1^. Unmanaged pain can have severe negative effects on ICU patients, particularly patients in critical conditions, triggering various mechanisms, such as increased catecholamine release, immune suppression, and elevated metabolic rates. These mechanisms can further lead to hemodynamic instability (e.g., elevated blood pressure and tachycardia), dyspnea, delirium, hyperglycemia, urinary retention, and cause different serious clinical consequences^2^. Continuous and effective pain assessment can not only help identify patient discomfort and guide analgesic treatment strategies promptly but also reduce the overuse of analgesic medications, thus promoting the recovery process of patients^3^.

Currently, the pain assessment process in clinical practice entirely relies on manual evaluation performed by healthcare professionals, which has several disadvantages. First, this method is characterized by observer bias. Namely, critically ill patients are often unable to self-report their pain, which requires using behavioral assessment scales, such as the critical care pain observation tool (CPOT)^4^. The judgment results obtained from different observers based on a patient’s behavior are highly subjective, making it challenging to achieve standardization and consistency in clinical practice. In the study conducted across eight ICUs in Scotland, the quality of sedation and analgesia was evaluated in 881 patients during 12-hour nursing periods, where only 56% of cases received high-quality sedation and analgesia^5^. Second, there is a significant consumption of human resources. For instance, in China, critical care staffing is in short supply. In the UK, each ICU bed requires at least seven nursing staff, whereas in China, even with the required nurse-to-bed ratio of 1:2.5–3, the majority of hospitals fail to meet this standard^6^. Third, the frequency of necessary assessments is limited, and continuous monitoring is not feasible. The pain of ICU patients can change over time depending on their conditions. However, the current pain assessment model is discontinuous, often resulting in delayed assessments. Recent studies have shown that 43.3% of ICU units assess sedation and analgesia less frequently than every four hours^7^. Therefore, developing an automated, non-contact pain assessment system has become an urgent need in the field of ICU clinical care. Such a system should be able to monitor and quantify patients’ pain levels in real-time, without relying on subjective judgments from healthcare providers. In addition, it should provide a basis for precise pain management, improve management efficiency, reduce the burden on medical staff, and promote the advancement of critical care toward a more intelligent and patient-centered direction.

**Fig. 1:**
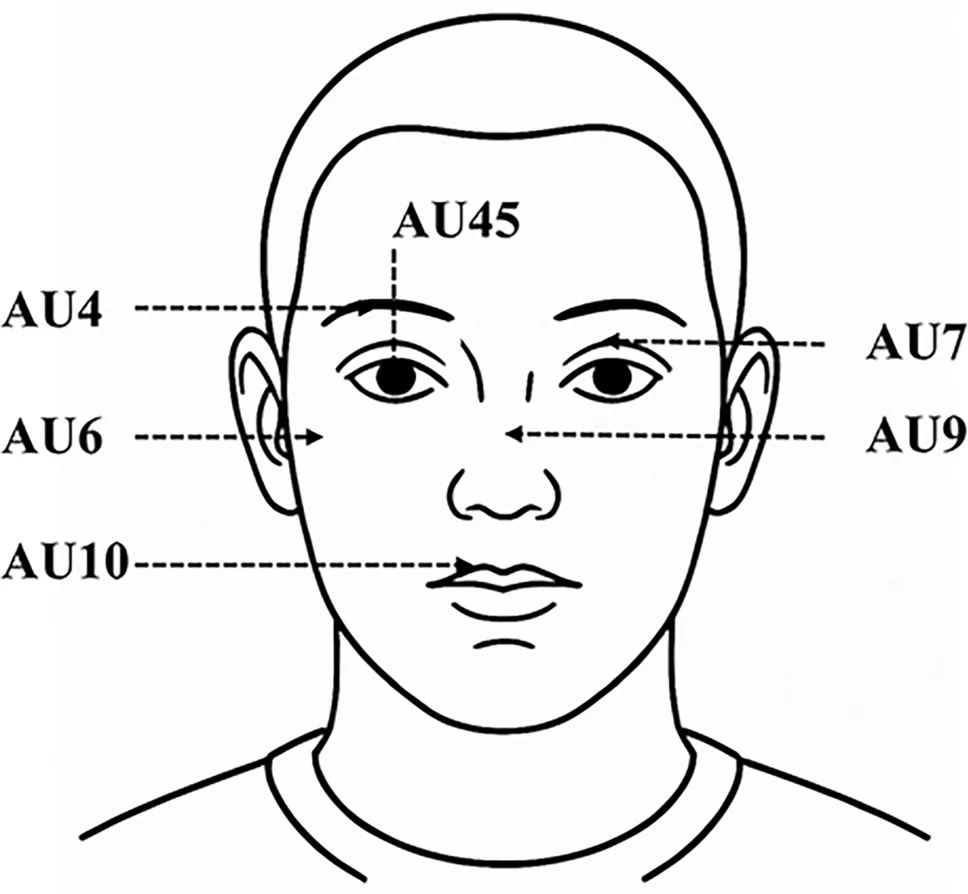
Visualization of the PSPI-related AU locations.

Facial expressions denote one of the most instinctive and immediate responses to pain, which makes them a promising avenue for automatic pain assessment^8–11^. The facial action coding system (FACS), which was primarily developed to quantify facial muscle movements through action units (AUs), provides a structured framework for pain analysis^12^. Over the past two decades, researchers have leveraged the FACS, particularly through tools like OpenFace^13^, aiming to develop automated systems to detect pain-related AUs from a facial video. One of the most widely used pain indices based on facial expression is the Prkachin and Solomon pain intensity (PSPI) metric^8^, which sums the intensities of several units, including AU 4 (brow lowering), AU 6 (cheek raising), AU 7 (lid tightening), AU 9 (nose wrinkling), AU 10 (upper lip raising), and AU 45 (blinking), as depicted in Fig. 1. These AUs have been generally regarded as prototypical facial features associated with pain expression in healthy individuals under controlled laboratory conditions. They have also been extensively employed in recent studies as input features for both traditional classifiers and deep learning-based models aimed at developing automated pain recognition systems. For instance, Sikka et al^14^. used the AUs extracted by the iMotions platform to detect postoperative pain in children. Xu et al^15^. proposed a multi-task, multi-dimensional model based on the PSPI-related AUs, which can directly predict visual analog scale scores from video sequences. Cascella et al^16^.constructed a binary classifier model using facial AUs for binary pain assessment in cancer patients. Zhanli Chen et al^17^. extracted frame-level AUs and adopted frameworks based on multiple-instance learning and multi-cluster instance learning to capture pain-relevant AU combinations from video data. Yuan et al^18^. further leveraged the AUs to guide facial texture modeling for pain recognition in patients with partially occluded faces.

**Fig. 2:**
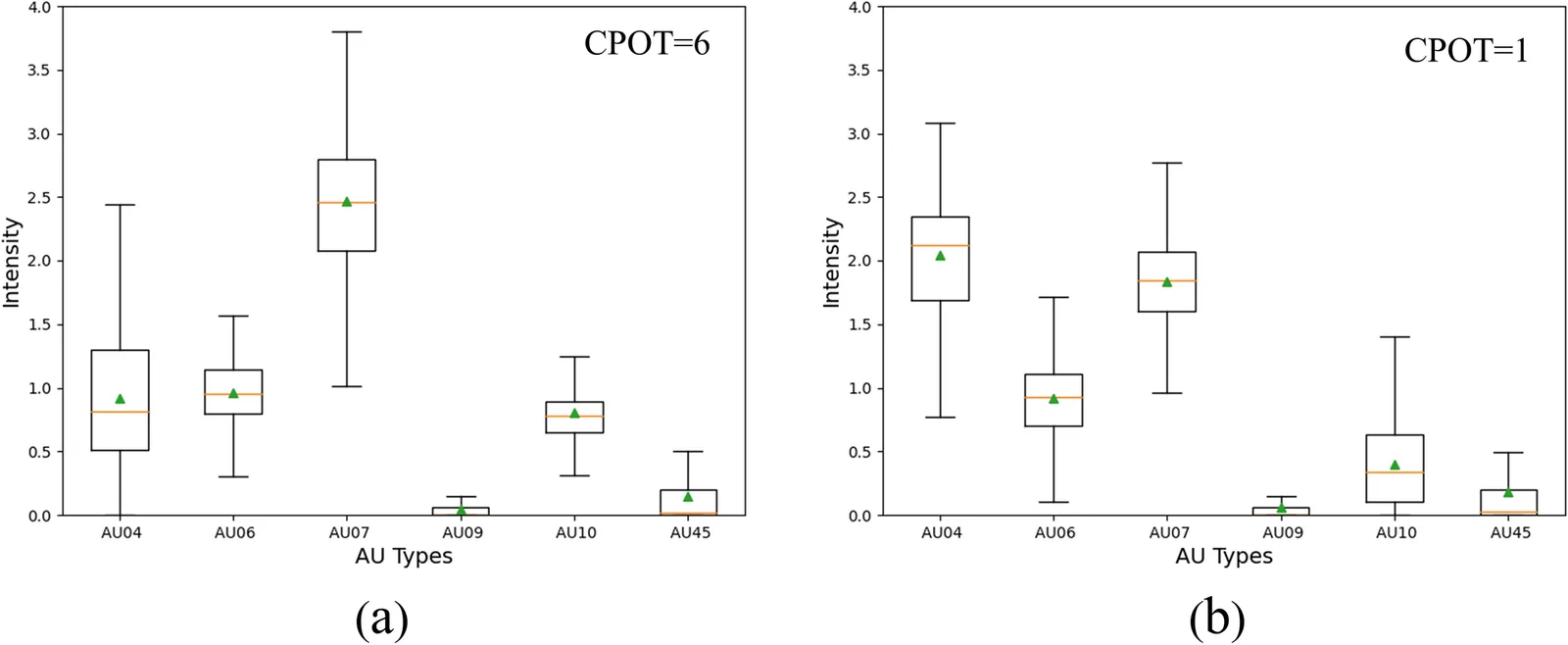
Statistical analysis of PSPI-related AUs for different facial pain expressions of two ICU patients; (b) an elderly patient having mild pain. The CPOT provides a validated score reflecting the patient’s current pain level based on the patient’s behavioral indicators.

However, translating recent research advances into real-world ICU clinical practice poses significant challenges^19^. First, ICU patients span a broad age range, where older individuals can exhibit more pronounced facial wrinkles and altered baseline muscle tone, which can obscure pain-related facial expressions and, thus, affect the pain detection results. Second, prolonged sedation, facial edema, and underlying neurological disorders can further compromise the clarity or presence of facial pain indicators^20^. Moreover, pain is inherently subjective, with individual differences in the pain threshold and intensity; also, cultural backgrounds and medical histories can significantly shape facial pain responses^21^. All the mentioned factors can affect the generalization ability of AU combinations derived from controlled experimental settings. As illustrated in Fig. 2, the PSPI-related AUs can appear similar in a young patient experiencing severe pain and an elderly patient experiencing mild discomfort, which highlights the limitations of conventional AU-based assessments in ICU clinical populations.

**Fig. 3:**
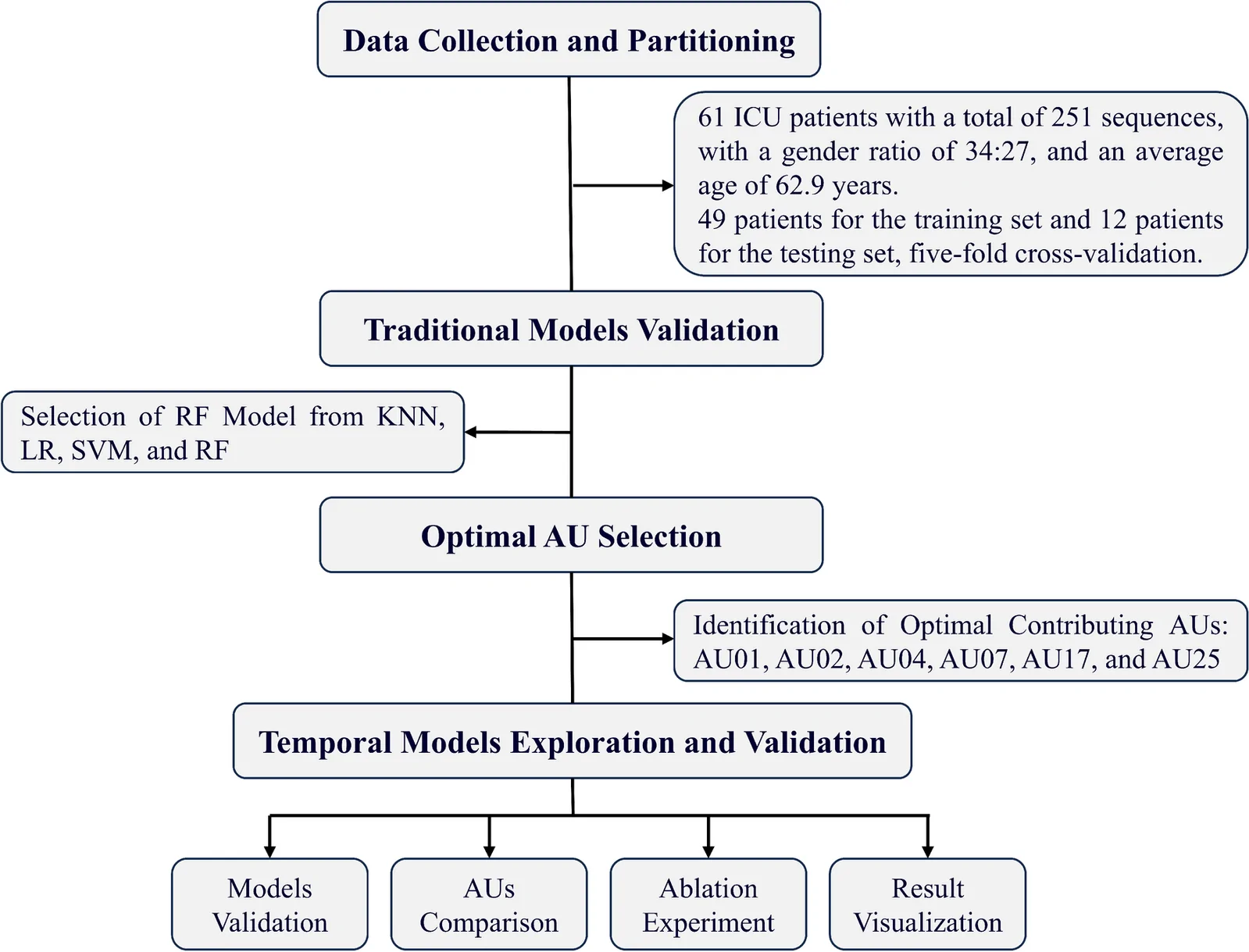
The overall framework of this study.

This study aims to bridge the gap between experimental research and clinical practice by developing a more accurate and robust method for extracting facial AU features tailored to ICU pain recognition systems. Our findings challenge the prevailing reliance on fixed AU combinations associated with the PSPI metric, offering new perspectives for future research in facial pain recognition. The overall framework of this study is shown in Fig. 3.

## Methods

### Data collection

The research conducted in this study was performed in strict accordance with ethical standards for clinical research. The data collection process was carried out at the Department of Critical Care Medicine at Beijing Jishuitan Hospital, Capital Medical University. The study protocol was reviewed and approved by the hospital’s Ethics Committee (Approval No. K2024-166-00) and adhered to the principles of the Declaration of Helsinki^22^. A convenience sampling method was employed to recruit patients admitted to the ICU of Beijing Jishuitan Hospital. The inclusion criteria were as follows: (1) age over 18 years, (2) postoperative ICU admission without endotracheal intubation, and (3) ability to express pain either verbally or nonverbally (e.g., via eye contact or gestures). However, patients were excluded from the study if they met any of the following conditions: (1) inability or unwillingness to report pain, (2) limb paralysis, impaired muscle function, deep sedation (Richmond Agitation-Sedation Scale score of up to –3), or use of neuromuscular blocking agents, (3) facial trauma or burns, facial dressings, facial paralysis, or any other condition significantly impeding facial image acquisition, and (4) a history of dementia, psychiatric disorders, or a clinical diagnosis of delirium.

This study adopted a prospective observational design. All enrolled patients participated voluntarily after signing informed consent forms; the forms were co-signed by both the patient and their legal guardian. In addition, a standardized video acquisition system was deployed using a modular trolley equipped with a 1080-pixel medical-grade camera, positioned approximately 1 m vertically above a patient’s nasal tip. To ensure data quality, the acquisition protocol included the following procedures: (1) maintaining consistent illumination during the data recording process to eliminate ambient light interference, (2) instructing patients to maintain a direct gaze toward the camera, and (3) initiating synchronous video capture while clinical staff performed concurrent pain assessments. We selected a 60-second recording window to balance data validity with clinical feasibility. This duration is long enough to capture both transient and sustained pain expressions while avoiding fluctuations caused by patient movement or changing conditions. It also minimizes patient burden and ensures stable, natural facial expressions for reliable analysis. The resulting dataset consisted of standardized video recordings from 61 patients, where each sequence had a resolution of 1,280 *×* 720 pixels, a frame rate of 20 frames per second, and a duration of 60 s. The study cohort consisted exclusively of individuals of Asian descent, with 34 males and 27 females and a mean age of 62.93 years (range: 24–89 years). They were all postoperative patients. The data from each enrolled patient includes at least one instance of resting (pain-free) data and three instances of data collected during natural or procedural clinical states. If the patient’s pain expression is significant, multiple recordings will be made, not exceeding six times. These data were collected between 8:00 AM and 6:00 PM. All collected data were encrypted and stored on the hospital’s local server, and data access was restricted through role-based permission control.

**Fig. 4:**
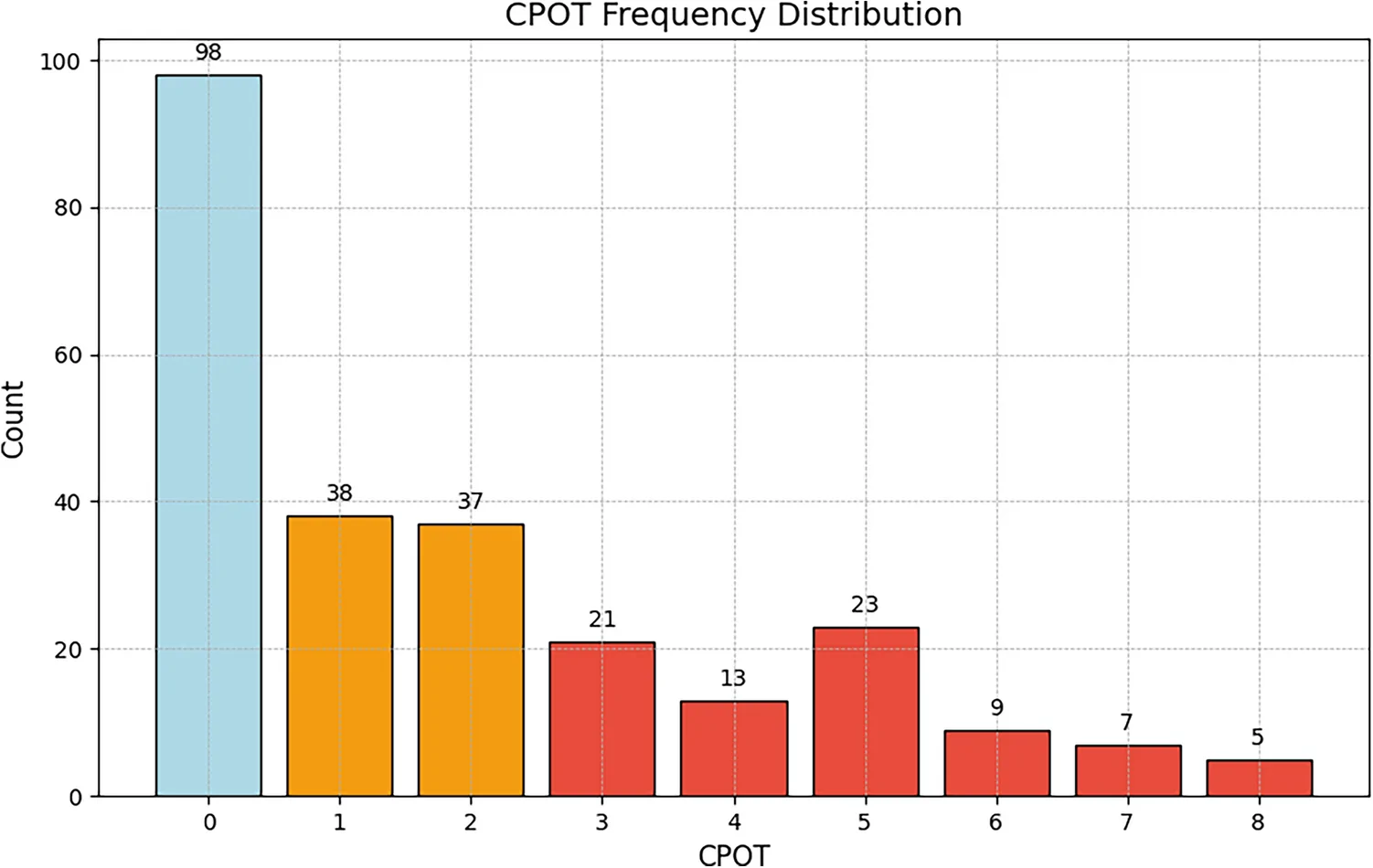
Statistics of patients’ CPOT scores. Blue represents painlessness, orange represents mild pain, and red represents severe pain.

Pain annotation was performed by an ICU nurse and a physician who had completed a standardized 36-hour training program aligned with the Guidelines for Pain Management in Critically Ill Patients^23^. The training process included modules on pain pathophysiology, micro-expression recognition, and consistency in procedural operations and was followed by a clinical case-based competence assessment. Dynamic pain evaluation was conducted using the critical-care pain observation tool (CPOT), which assessed four dimensions: facial expression, body movements, muscle tension, and vocalization. Each dimension was rated on a three-point scale, yielding a total score ranging from zero to eight. Each recording will involve CPOT scoring conducted by medical staff on-site. In total, 251 annotated samples were collected from 61 ICU patients. The consistency between the nurse and the doctor, as measured by Cohen’s kappa, was 0.86. In accordance with the ICU pain management guidelines, a score of three or greater indicated severe pain, a score of 1–2 meant mild pain, and a score of zero denoted no pain. Accordingly, two classification tasks were performed: no pain versus mild pain (CL1) and no pain versus severe pain (CL2). To ensure annotation reliability, samples exhibiting scoring discrepancies or inconsistencies were reviewed and re-annotated by a senior ICU attending physician with over 10 years of clinical experience. The final CPOT score distribution across the dataset is illustrated in Fig. 4. The class distribution for our tasks includes 98 BL, 75 CL1, and 78 CL2 samples. To minimize the potential effects of class imbalance, we intentionally collected a relatively balanced dataset.

### Pain assessment

To extract detailed information on facial AUs, this study employed OpenFace, an open-source toolkit that has been widely used in behavioral computing and affective analysis. The OpenFace toolkit was selected to be used in this study based on prior research. Seventeen facial AUs were framewise extracted, including AU01, AU02, AU04, AU05, AU06, AU07, AU09, AU10, AU12, AU14, AU15, AU17, AU20, AU23, AU25, AU26, and AU45. The AU intensity values range from 0 to 5. Further, to comprehensively characterize the distributional properties and temporal dynamics of the AU sequences, this study computed a broad range of statistical features. Specifically, for each AU, classical descriptive statistics, including the mean, median, standard deviation, maximum, minimum, range, first and third quartiles (Q1, Q3), interquartile range (IQR), skewness, and kurtosis values, were calculated. In addition, the first-order derivatives along the temporal axis were determined to capture dynamic changes in the AU intensity value. The same set of statistical descriptors was extracted from the derivative series. In total, 22 statistical features were aggregated into a high-dimensional feature vector, which provided rich input data for subsequent modeling and classification tasks.

To evaluate the suitability of different models in facial action unit-based pain recognition, this study selected four widely used supervised learning algorithms: random forest^24^, logistic regression^25^, support vector machine^26^, and K-nearest neighbors^27^ methods. These methods contained different algorithmic paradigms, including tree-based ensemble methods, linear classifiers, kernel-based margin maximizers, and distance-based voting schemes. Using a unified data partitioning scheme and consistent evaluation protocols, this study trained and tested four representative machine learning models to investigate which method is best suited for constructing a facial expression-driven pain recognition system.

**Random Forest**

The Random Forest (RF) is an ensemble learning method that aggregates predictions from multiple decision trees. The final prediction in the RF method is made through majority voting across all trees, which can be expressed as follows:

$$\hat{y} = \text {majority\_vote} \left\{ h_1(x), h_2(x), \ldots , h_t(x) \right\} ,$$

where $h_t(x)$ denotes the prediction from the *t*th decision tree for an input sample *x*.

**Logistic Regression**

The Logistic Regression (LR) method models the probability of a binary outcome using the sigmoid function over a linear combination of input features, which can be expressed as follows:

$$P(y = 1 \mid x) = \frac{1}{1 + e^{-(w^T x + b)}},$$

where *w* and *b* are model parameters learned from the data.

**Support Vector Machine**

The Support Vector Machine (SVM) method constructs a decision boundary that maximizes the margin between classes. With the radial basis function kernel, the decision function can be expressed as follows:

$$f(x) = \sum _{i=1}^{N} \alpha _i y_i K(x_i, x) + b,$$

$$K(x_i, x) = \exp (-\gamma \Vert x_i - x\Vert ^2),$$

where *f*(*x*) is the decision function, $\alpha _i$ are the support vector coefficients, $y_i$ are the labels of the training samples, $K(x_i, x)$ is the kernel function, *b* is the bias term, *γ* is the kernel parameter, and *N* is the number of training samples.

**K-nearest Neighbors**

The K-nearest Neighbors (KNN) method classifies a sample by taking the majority vote of its *k* nearest training samples based on a distance metric as follows:

$$\hat{y} = \text {majority\_vote} \left\{ y_i \mid x_i \in \mathscr {N}_k(x) \right\} ,$$

where $\mathscr {N}_k(x)$ is the set of the *k* nearest neighbors to a sample *x*.

To evaluate the four classical machine learning models, this study selected the feature importance metrics from the best-performing model to identify the facial AUs most relevant to pain recognition in ICU patients. The feature selection procedure was instrumental in eliminating redundant information and concentrating on the AU features that contributed most to the classification result. Further, to capture the temporal dynamics inherent in facial expressions, this study employed four advanced temporal modeling architectures to analyze the selected AU sequences: the recurrent neural network^28^, long short-term memory network^29^, temporal convolutional network^30^, and Transformer model^31^. The four selected models denote the mainstream paradigms based on recurrence, memory augmentation, convolutional hierarchy, and attention mechanisms. By systematically comparing the performance of the four models in AU sequence modeling, this study aimed to assess the suitability and effectiveness of different temporal learning strategies for pain recognition in ICU patients under real-world clinical conditions.

**Recurrent Neural Network**

The Recurrent Neural Network (RNN) model computes a hidden state $h_t$ as a nonlinear transformation of the current input $x_t$ and the previous hidden state $h_{t-1}$ as follows:

$$h_t = \tanh (W_{xh}x_t + W_{hh}h_{t-1} + b).$$

where $h_t$ is the hidden state at time step *t*, $x_t$ is the input at time step *t*, $h_{t-1}$ is the hidden state from the previous time step, $W_{xh}$ is the weight matrix for the input to hidden state, $W_{hh}$ is the weight matrix for the hidden state to hidden state, and *b* is the bias term.

**Long Short-term Memory**

The Long Short-term Memory (LSTM) method adopts memory cells and gating mechanisms to address the vanishing gradient problem and includes the following computations:

$$\begin{aligned} f_t&= \sigma (W_f x_t + U_f h_{t-1} + b_f), \\ i_t&= \sigma (W_i x_t + U_i h_{t-1} + b_i), \\ o_t&= \sigma (W_o x_t + U_o h_{t-1} + b_o), \\ c_t&= f_t \odot c_{t-1} + i_t \odot \tanh (W_c x_t + U_c h_{t-1} + b_c), \\ h_t&= o_t \odot \tanh (c_t). \end{aligned}$$

where $f_t$ is the forget gate output at time step *t*, $i_t$ is the input gate output at time step *t*, $o_t$ is the output gate output at time step *t*, $c_t$ is the cell state at time step *t*, $h_t$ is the hidden state at time step *t*, $x_t$ is the input at time step *t*, *W*, *U*, and *b* are the weight matrices and biases for different gates, *σ* is the sigmoid activation function, *⊙* denotes element-wise multiplication, and *\tanh* is the hyperbolic tangent activation function.

**Temporal Convolutional Network**

The Temporal Convolutional Network (TCN) model employs dilated convolutions to expand the receptive field over time without increasing the model depth. The one-dimensional dilated convolution is defined as follows:

$$y(t) = \sum _{i=0}^{k-1} f(i) \cdot x(t - d \cdot i),$$

where *d* is the dilation factor, and *k* is the filter size.

**Transformer Model**

The Transformer architecture employs a self-attention mechanism to capture long-range dependencies across the input sequence, which is defined as follows:

$$\text {Attention}(Q, K, V) = \text {softmax} \left( \frac{QK^T}{\sqrt{d_k}} \right) V,$$

where *Q*, *K*, and *V* denote the query, key, and value matrices, respectively, and $d_k$ is the dimensionality of the key vectors.

## Experimental results

### Implementation details

In this study, We randomly divided the 61 patients into five groups based on their medical record numbers. Since 61 is not divisible by 5, the remaining one patient was included in the training set. Therefore, each fold consists of data from 49 patients for training and 12 patients for testing. All network structures is built using the PyTorch deep learning library. All experimental training and testing are conducted on a DELL workstation equipped with a Dell Xeon Gold 6226R CPU and an NVIDIA RTX 3090 GPU. In the traditional model phase, we used Random Forest with 40 estimators, Logistic Regression with 2000 iterations, SVM (RBF kernel) with C=10, gamma=‘scale’, and balanced class weights, and KNN with 10 neighbors. In the deep learning model phase, we selected the Adam optimizer, commonly used in the field, with an initial learning rate of 0.0001 and a batch size of 32. For all experiments, the number of training epochs was fixed at 64.

### Model validation results

To evaluate the suitability of the four supervised learning models for pain recognition driven by facial expressions in ICU patients, this study conducted two binary classification tasks based on the CPOT scoring system. Task CL1 involved distinguishing “no pain” (CPOT score = 0) from “mild pain” (CPOT score = 1–2), whereas task CL2 focused on distinguishing “no pain” from “severe pain” (CPOT score *≥* 3). The input features consisted of 22 statistical descriptors derived from 17 facial AUs, and four widely used classification algorithms were applied: the KNN, LR, SVM, and RF models. We have reviewed numerous works in the field of facial pain assessment, all of which adopted five-fold cross-validation as their evaluation protocol^32–34^. Consequently, we chose to follow this established evaluation method. The evaluation metrics included accuracy, precision, recall, and F1-score. The comparison results are summarized in Table 1.

**Table 1:** Performance comparison of the four supervised learning models across two classification tasks: CL1 (no pain vs. mild pain) and CL2 (no pain vs. severe pain). Each metric is presented in the form of (mean ± standard deviation) over the five-fold cross-validation. Bold indicates the best result. () denotes the p-value from a five-fold paired t-test between this model and RF.

**Task**	**Model**	**Accuracy**	**Precision**	**Recall**	**F1-score**
CL1	KNN	57.80±9.35 (0.197)	57.68±10.37 (<0.001)	56.18±8.57 (0.002)	55.80±8.73 (0.066)
	LR	54.40±6.18 (<0.001)	53.61±5.87 (<0.001)	53.18±5.78 (<0.001)	52.57±5.81 (0.006)
	SVM	55.50±5.83 (<0.001)	54.25±5.64 (<0.001)	53.66±5.52 (0.001)	52.88±5.81 (0.001)
	**RF**	**59.55±3.04**	**58.24±4.00**	**56.79±3.22**	**55.94±3.58**
CL2	KNN	63.56±11.21 (<0.001)	64.00±12.38 (0.007)	61.48±11.27 (<0.001)	60.90±11.62 (<0.001)
	LR	73.24±11.31 (<0.001)	73.31±11.86 (<0.001)	73.26±12.11 (<0.001)	72.83±11.70 (<0.001)
	SVM	65.32±5.05 (<0.001)	66.86±7.07 (0.001)	65.81±6.09 (<0.001)	64.75±5.34 (0.009)
	**RF**	**76.06±8.30**	**77.56±8.69**	**75.98±8.89**	**75.35±8.75**

**Fig. 5:**
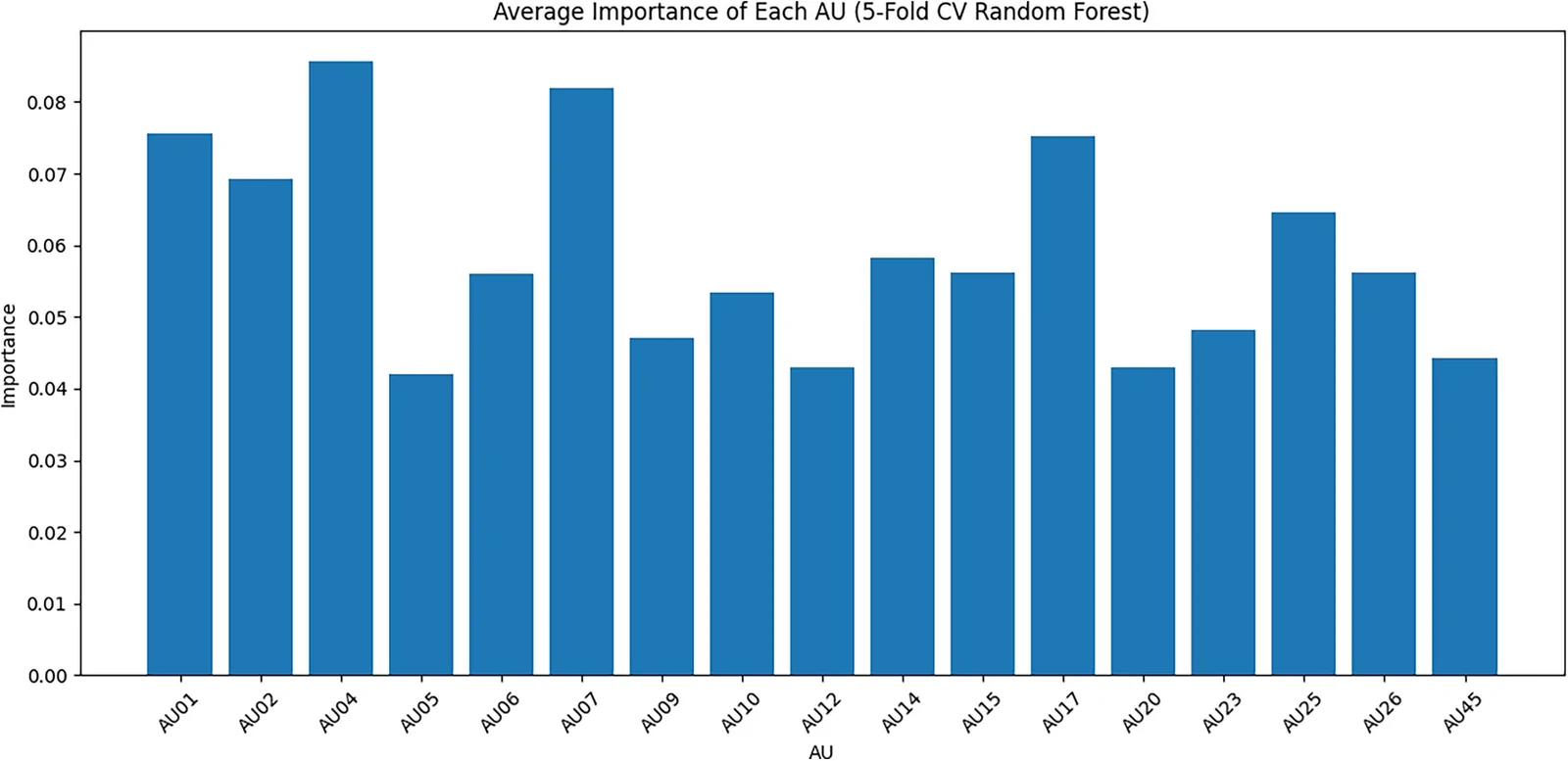
Feature importance of individual AUs in the CL1 classification task using the RF model with five-fold cross-validation.

In the CL1 task, the RF model outperformed all other baselines across all metrics, achieving an accuracy of 59.55%, a precision of 58.24%, a recall of 56.79%, and an F1-score of 55.94%. These results indicated superior performance of the RF model compared to the other models. The LR and SVM models exhibited similar but lower performance compared to the RF model, suggesting that the subtle facial differences between “no pain” and “mild pain” could not be easily captured by linear or kernel-based methods. In contrast, the overall classification performance improved significantly in the CL2 task, indicating that facial AUs under severe pain conditions exhibited stronger discriminative patterns. Again, the RF model achieved the highest performance among all the models, with an accuracy of 76.06%, a precision of 77.56%, a recall of 75.98%, and an F1-score of 75.35%, thus outperforming both the LR (accuracy = 73.24%) and SVM (accuracy = 65.32%) models. These findings further highlighted the robustness and generalization ability of the RF model in modeling high-dimensional and nonlinear facial features. In contrast, although the KNN model showed no statistically significant difference from RF in terms of accuracy in the CL1 task, its performance was clearly inferior in the CL2 task. This may be partly attributed to its sensitivity to sample distribution and outliers, as well as its limitations in capturing complex feature interactions. Consequently, the comparison results demonstrated that the RF model consistently achieved superior performance across different pain recognition tasks, demonstrating strong potential for building reliable ICU pain assessment systems based on facial AU analysis.

### Optimal AU selection

**Fig. 6:**
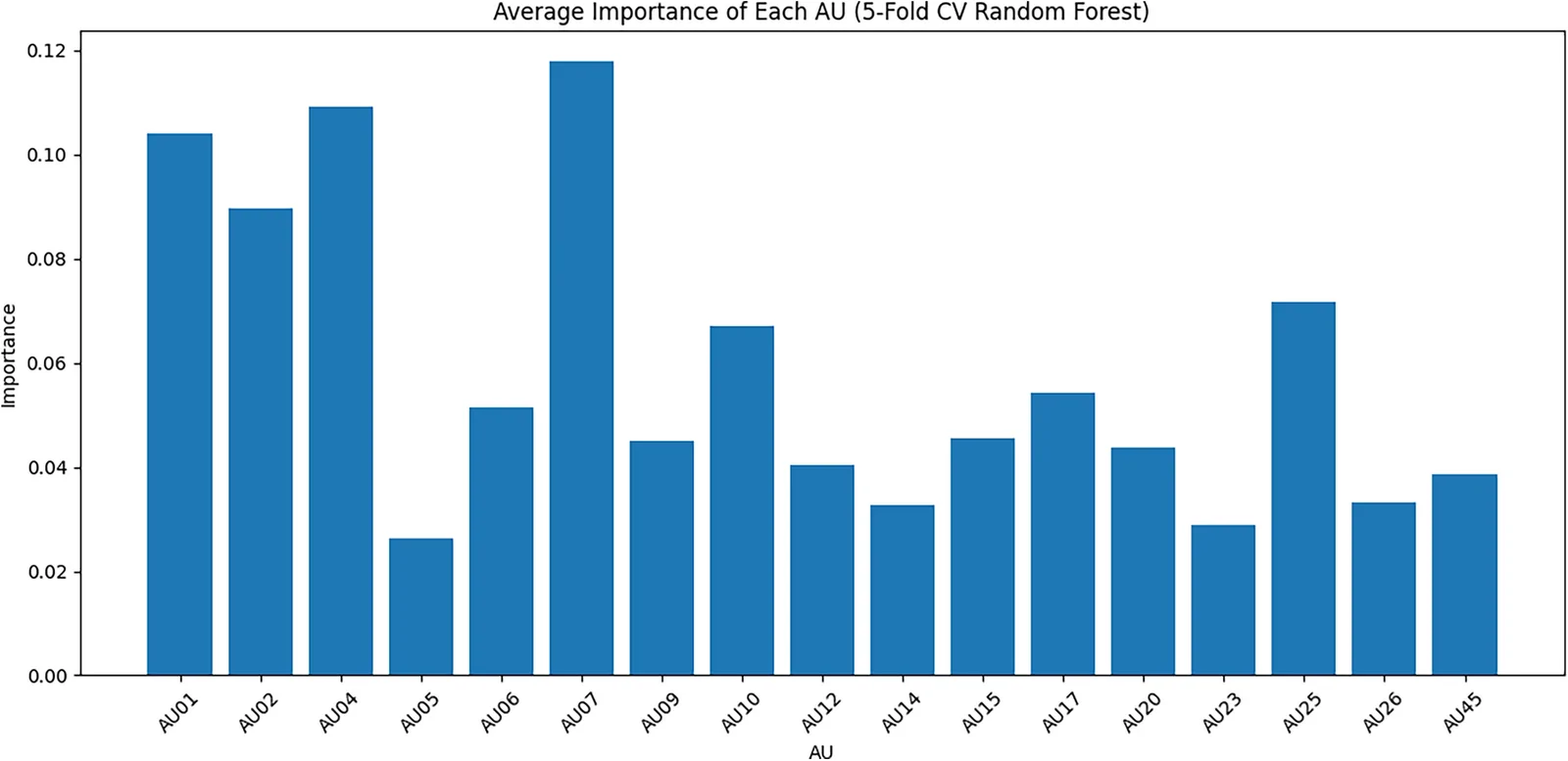
Feature importance of individual AUs in the CL2 classification task using the RF model with five-fold cross-validation.

After evaluating the four classical machine learning-based models, this study further used the best-performing model, namely the RF model, to conduct a feature importance analysis of the input facial action units, aiming to identify the most informative features for pain recognition. In two binary classification tasks, the CL1 (no pain vs. mild pain) and CL2 (no pain vs. severe pain) tasks, the average importance score was computed for each AU through five-fold cross-validation. The calculation results are presented in Figs. 5 and 6. In the CL1 task, the top five AUs ranked by importance were as follows: AU04 (brow lowering), AU07 (lid tightening), AU01 (inner brow raiser), AU17 (chin raiser), and AU02 (outer brow raiser); this indicated that these features exhibited strong discriminative capacity in distinguishing mild pain states. In contrast, the CL2 task identified AU07, AU04, AU01, AU02, and AU25 (lips part) as the most significant AUs, suggesting that in case of severe pain, muscle activity around the eyes, brows, and mouth became more pronounced and thus more readily captured by the model. By integrating the importance rankings from the two tasks, this study ultimately selected six AUs that consistently demonstrated high relevance, namely AU01, AU02, AU04, AU07, AU17, and AU25, and used them as key input features for subsequent pain recognition modeling.

**Table 2:** Performance comparison of different AU feature strategies in the RF classification tasks (mean ± std). () denotes the p-value from a five-fold paired t-test between this feature and Ours.

**Task**	**AU Features**	**Accuracy**	**Precision**	**Recall**	**F1-score**
CL1	All	59.55±3.04 (0.003)	58.24±4.00 (0.008)	56.79±3.22 (<0.001)	55.94±3.58 (<0.001)
	PSPI	60.05±6.54 (<0.001)	60.75±9.18 (0.089)	58.77±6.27 (<0.001)	57.76±6.44 (0.049)
	**Ours**	**61.28±4.22**	**61.38±4.86**	**60.32±4.18**	**59.73±3.95**
CL2	All	76.06±8.30 (0.006)	77.56±8.69 (<0.001)	75.98±8.89 (0.005)	75.35±8.75 (<0.001)
	PSPI	76.65±9.19 (0.014)	77.11±9.75 (<0.001)	76.23±9.83 (0.002)	75.96±9.75 (0.003)
	**Ours**	**78.37±6.25**	**80.52±7.14**	**78.20±7.15**	**77.57±6.73**

This study further compared the performance of three AU input strategies in the RF model: (1) using all available AUs (All), (2) using PSPI-related AUs (PSPI), and (3) using the important AUs identified by us (Ours). As shown in Table 2, the AU subset selected by the proposed method consistently outperformed those selected by the other two strategies across all evaluation metrics in both classification tasks. In the CL1 task, the AU combination achieved marginally higher accuracy, precision, and F1-score compared to the PSPI and full AU sets, which indicated improved feature compactness and discriminative capacity. In the CL2 task, the proposed method led to a marked enhancement in model performance, with an accuracy of 78.37%, a precision over 80%, and an F1-score of 77.57%, thus surpassing the benchmarks set by the other two strategies. These results demonstrated that the proposed AU selection method could effectively retain critical facial movement information while improving model robustness and generalization ability.

### AU dynamics temporal modeling analysis

**Fig. 7:**
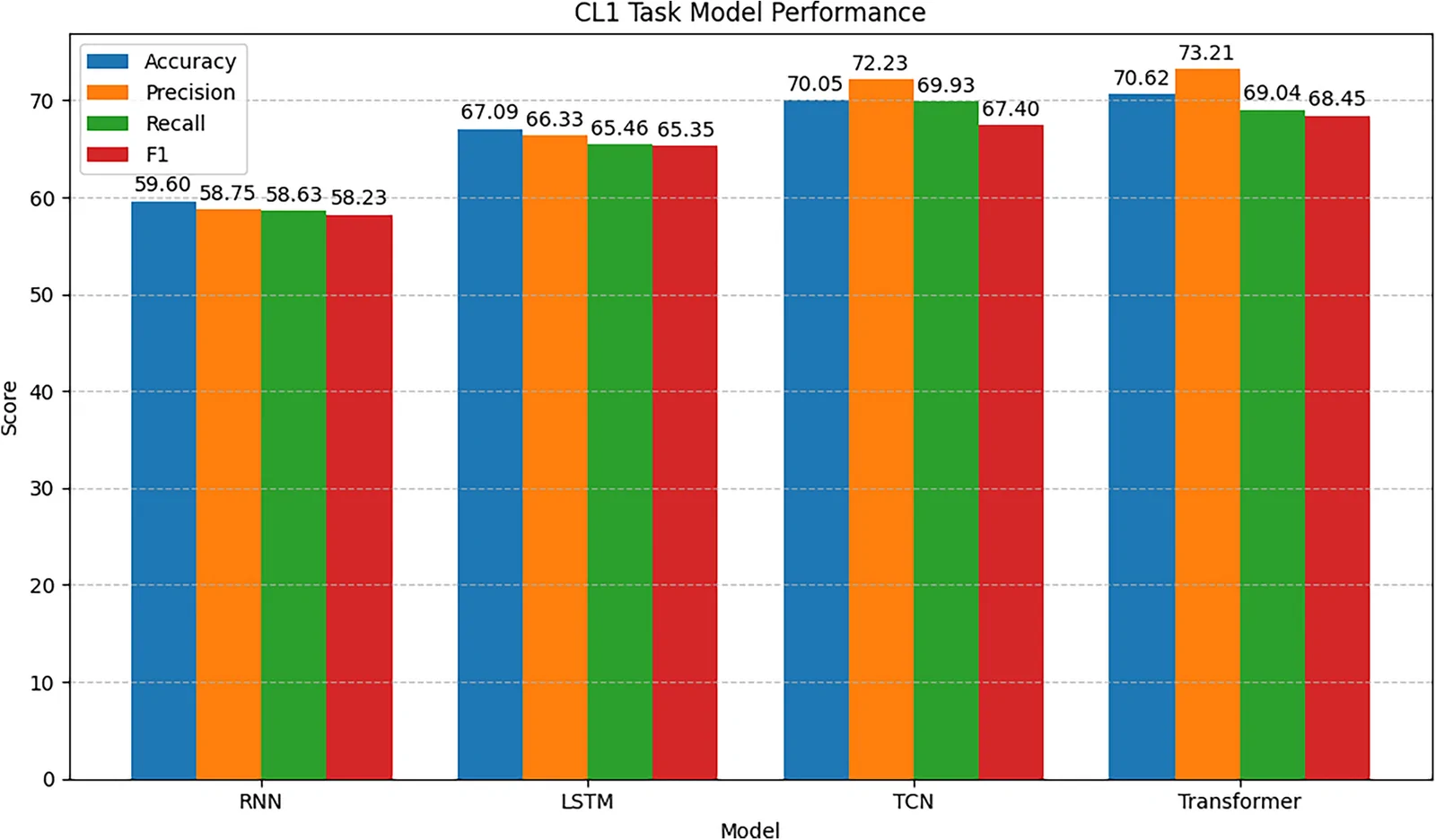
Performance of the optimal AU combination in the CL1 classification task for four temporal models.

**Fig. 8:**
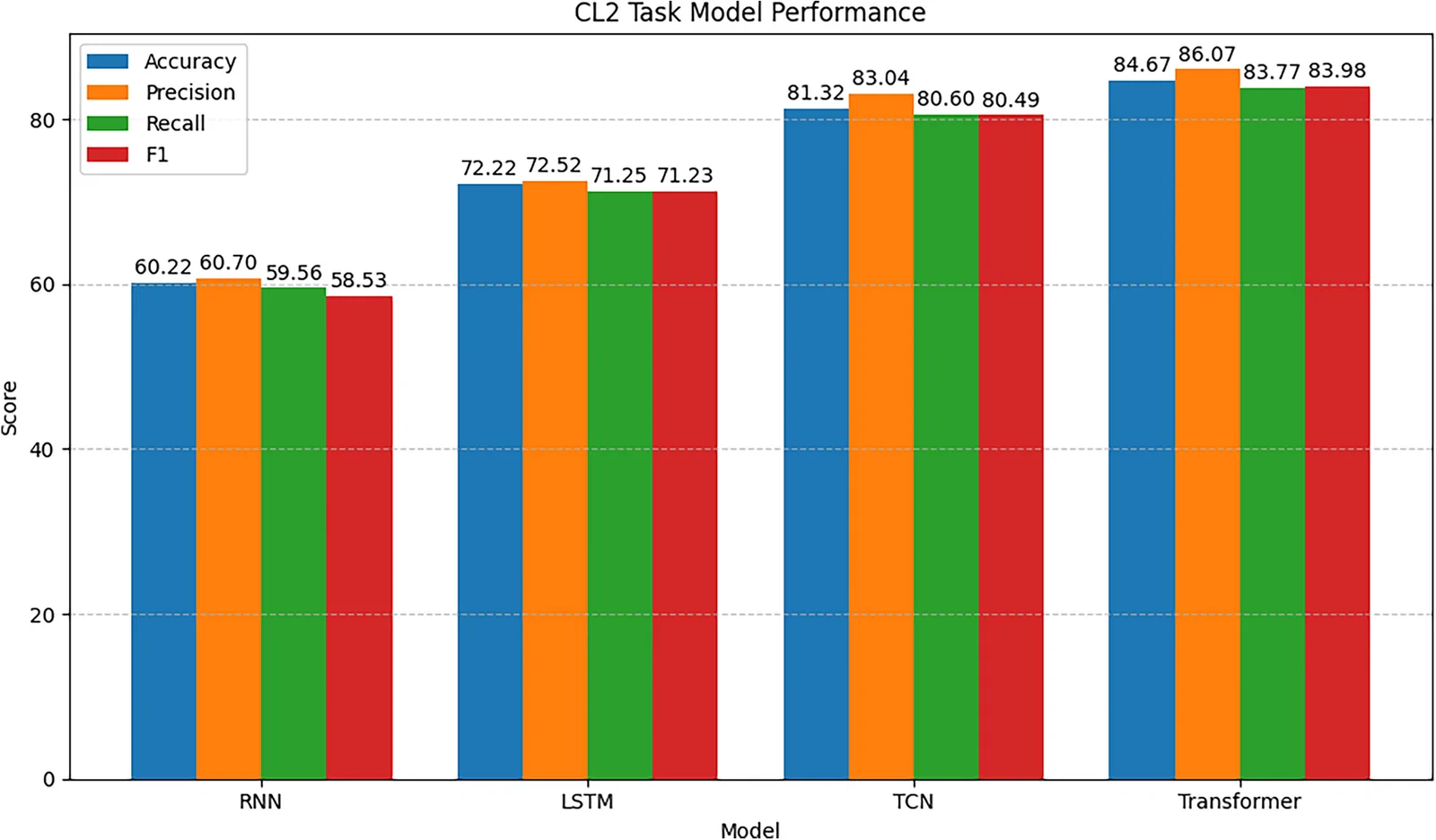
Performance of the optimal AU combination in the CL2 classification task across the four temporal models.

After identifying key AUs, this study further explored a temporal modeling approach for pain recognition based on dynamic AU sequences. Particularly, the manually engineered statistical features were replaced with raw AU time series of length 1200, and four representative deep learning-based architectures were employed for sequential modeling, namely RNN, LSTM, TCN, and Transformer. These models were used to autonomously learn the temporal evolution patterns of facial AUs. The experimental results are illustrated in Figs. 7 and 8. The results indicated that in the first classification task, which distinguished between no pain and mild pain, the Transformer model achieved the highest accuracy of 70.62% and an F1 score of 68.45% among all the models. This performance indicated a clear improvement over the traditional temporal models, as the RNN model yielded an F1 score of 58.23%, and the LSTM model achieved 65.35%. The advantage of the Transformer became even more prominent in the second classification task, which involved distinguishing between the no-pain and severe-pain scenarios. The Transformer reached an accuracy of 84.67% and an F1 score of 83.98%, thus outperforming the TCN model, which obtained an F1 score of 80.49%, and the LSTM model, which achieved 71.23%.

The superior performance of the Transformer architecture could be attributed to its self-attention mechanism, which enabled global contextual modeling of dependencies between any two time points in the input sequence. This capability allows the model to overcome limitations commonly encountered in traditional recurrent networks, such as vanishing gradients and difficulties in capturing long-range dependencies. In addition, the Transformer supported efficient parallel computation, which made it particularly suitable for large-scale and high-dimensional sequential data. Considering the inherent complexity of AU signals observed in real-world clinical environments, including nonlinear signal dynamics, asynchronous activations, and multi-scale temporal patterns, the multi-head attention mechanism employed in the Transformer offered a fine-grained representation of inter-AU coupling relationships. This design can enhance both the expressive capacity and model robustness.

**Fig. 9:**
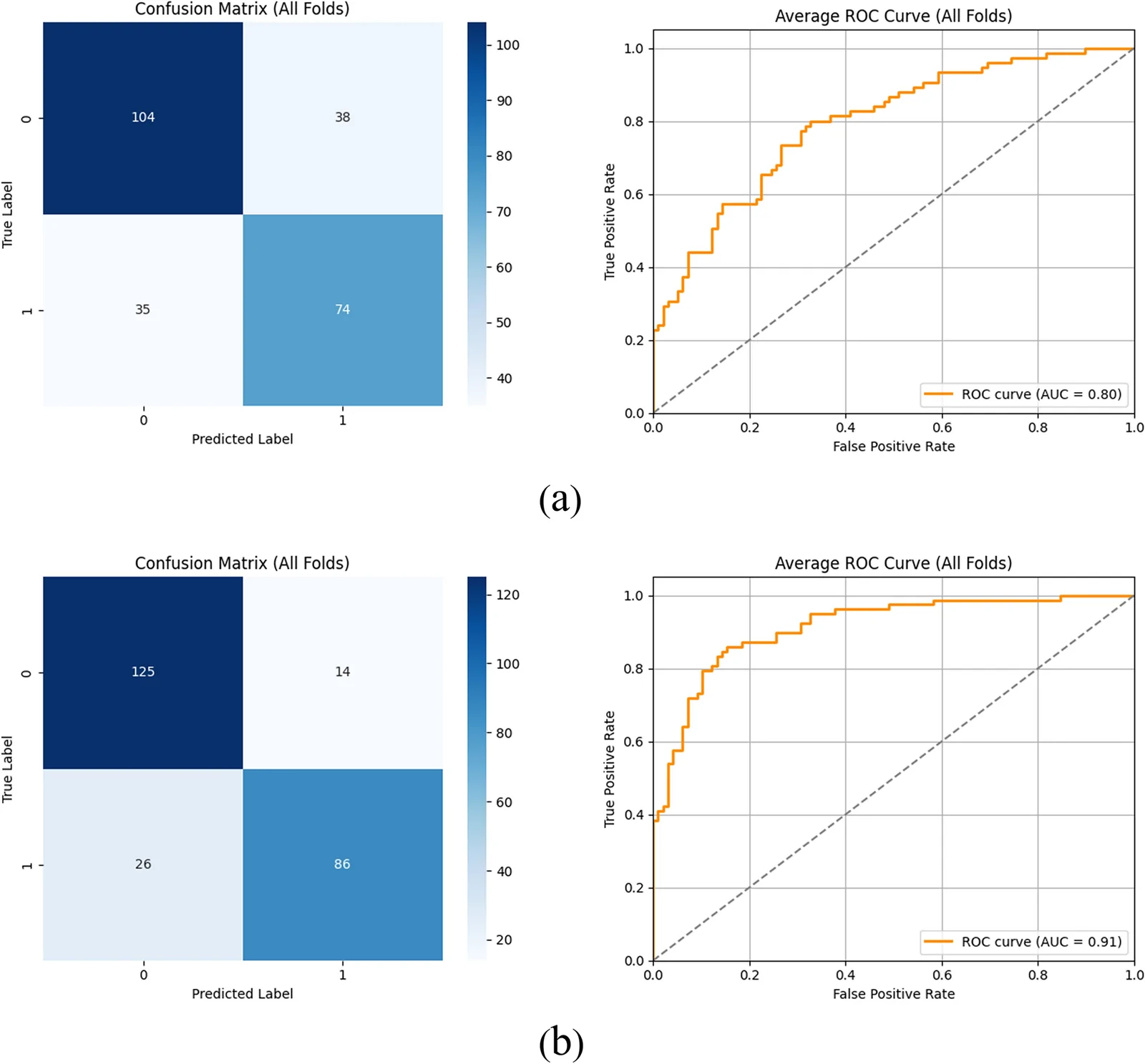
The confusion matrix and ROC curve for all folds of the Transformer on both tasks: (a) CL1 task, (b) CL2 task.

Subsequently, the predefined PSPI AU combination and the optimized AU combination were validated using the best-performing TCN and Transformer models; the experimental results are presented in Table 3. In the CL1 task, the TCN model with the proposed AU combination showed a slightly lower accuracy than the PSPI model but exhibited improvements in terms of precision and F1 score. Notably, the variance in precision using the PSPI configuration was particularly high, indicating its instability. In the case of the Transformer model, the accuracy and precision obtained using the proposed AU combination were both marginally higher than those of the PSPI; the F1-score also improved, highlighting the advantage of the proposed AU combination. In the CL2 task, the TCN model with the proposed AU combination outperformed the PSPI model in terms of accuracy, whereas the Transformer model demonstrated more substantial gains, with both accuracy and F1 score exceeding those of the PSPI model. These results indicated that the proposed AU combination could provide a advantage in recognizing severe pain. Therefore, the integration of temporal modeling with AU optimization strategies could significantly improve performance in facial pain recognition.The results in Table 4 show that removing AU4 from the model leads to a notable decrease in performance across all metrics, particularly in accuracy and F1-score. This suggests that AU4 plays a critical role in improving the model’s ability to recognize pain, highlighting its importance in the CL2 task. In addition, the visualization of the test results for all folds of the Transformer on both tasks is shown in Figure. 9.

**Table 3:** Performance comparison results of different AU feature strategies across the TCN and Transformer models (mean ± std). () denotes the p-value from a five-fold paired t-test between this feature and Ours.

**Task**	**Model**	**AU Features**	**Accuracy**	**Precision**	**Recall**	**F1-score**
CL1	TCN	PSPI	71.16±10.16 (0.169)	65.79±20.33 (<0.001)	68.65±11.90 (0.008)	66.15±16.68 (0.002)
		**Ours**	**70.05±9.47**	**72.23±8.90**	**69.93±11.04**	**67.40±12.77**
	Transformer	PSPI	69.39±11.86 (0.034)	70.92±12.80 (<0.001)	67.57±12.12 (<0.001)	66.82±12.61 (<0.001)
		**Ours**	**70.62±8.59**	**73.21±9.57**	**69.04±9.63**	**68.45±9.32**
CL2	TCN	PSPI	79.60±10.11 (<0.001)	82.21±9.44 (<0.001)	77.53±11.37 (0.035)	77.31±12.50 (<0.001)
		**Ours**	**81.32±6.59**	**83.04±7.26**	**80.06±7.28**	**80.49±7.18**
	Transformer	PSPI	81.25±5.57 (0.003)	83.55±8.47 (0.007)	80.06±6.18 (<0.001)	80.26±6.03 (0.002)
		**Ours**	**84.67±3.85**	**86.07±4.83**	**83.77±3.81**	**83.98±4.02**

**Table 4:** The ablation study results of the Transformer framework in the CL2 task (mean ± std).

**AU Features**	**Accuracy**	**Precision**	**Recall**	**F1**
Ours W/O AU1	81.29 ± 3.63	83.20 ± 2.19	80.69 ± 4.96	80.36 ± 4.98
Ours W/O AU2	80.14 ± 5.28	82.77 ± 4.64	78.73 ± 6.32	78.74 ± 6.30
Ours W/O AU4	75.59 ± 2.01	80.24 ± 3.81	74.61 ± 3.68	73.78 ± 3.61
Ours W/O AU7	78.94 ± 7.21	79.39 ± 7.97	77.89 ± 7.77	78.08 ± 7.94
Ours W/O AU17	83.51 ± 5.58	85.11 ± 5.88	82.94 ± 5.90	82.91 ± 5.85
Ours W/O AU25	81.24 ± 5.63	83.78 ± 6.73	80.77 ± 5.87	80.53 ± 5.72
**Ours**	**84.67 ± 3.85**	**86.07 ± 4.83**	**83.77 ± 3.81**	**83.98 ± 4.02**

## Discussion

This study investigates the potential of facial AUs in pain state recognition, specifically in ICU patients, performing a comprehensive evaluation across feature selection, classical machine learning, and deep temporal modeling strategies. The results of the experiments conducted with the four widely used supervised-learning models demonstrated that the RF classifier consistently achieved superior performance in both classification tasks. This could be attributed to its robustness in modeling high-dimensional and nonlinear features. Further, to identify the most discriminative AU features, this study conducted feature importance analysis using the RF framework and six key AUs, namely AU01, AU02, AU04, AU07, AU17, and AU25. The results indicate that the data-driven subset outperforms both the complete AU set and the predefined PSPI AU set in classification performance, supporting the value of automatic AU selection in ICU clinical facial analysis. Using the selected AUs, this study further explored temporal characteristics by comparing four popular sequential models, namely the RNN, LSTM, TCN, and Transformer models, using raw AU sequences as model input. The results showed that the Transformer model outperformed all other models in both tasks, achieving an F1-score of 83.98% in the CL2 task. This performance improvement could be attributed to the self-attention mechanism, which enabled the Transformer to capture long-range dependencies and asynchronous activations between AUs, further enhancing the model’s sensitivity to subtle temporal changes in facial expression.

Currently, mainstream facial pain assessment methods commonly applied to publicly available datasets often rely on specific AUs selected by the PSPI to quantify pain features. However, due to its dependence on a fixed AU combination, the PSPI faces some limitations in clinical applications. Namely, in clinical settings, factors such as the patient’s condition type, disease stage, age structure, and individual facial characteristics can interfere with facial expression patterns, thus affecting the accuracy and generalization ability of PSPI indicators. The fixed combinations designed for healthy populations may not adequately address the needs of ICU patients. Therefore, ensuring dynamical learning of the pain representation features for different clinical scenarios and effectively capturing the relationship between environmental, individual, and facial expression changes denotes a critical direction for improving the clinical adaptability of pain recognition systems.

This study represents a preliminary exploration of this complex issue. We believe that the findings of this research are not intended to completely replace manual assessments in clinical settings, but rather to facilitate the early detection of potential pain, providing alerts for nurses and documenting pain trends to assist physicians in reviewing and adjusting treatment strategies. Through ongoing technological improvements, we aim to reduce false positives and false negatives. For instance, first, the adoption of more refined AU intensity estimation or micro-expression detection techniques could enhance sensitivity to subtle or ambiguous pain signals. Second, the development of proprietary models to accommodate the facial textures and personalized pain expressions of ICU patients is underway. Finally, integrating facial expression data with vital signs^35^ (such as blood pressure, heart rate, and oxygen saturation) and electronic health records will enable the construction of a more comprehensive and robust pain assessment system.

In conclusion, the combination of optimized AU feature selection and deep temporal modeling has been proven to be effective in enhancing automatic pain recognition performance. The results presented in this work provide valuable insights into the development of non-invasive and intelligent pain assessment frameworks, with strong potential for deployment in clinical practice.

## Limitations

This study has several limitations that should be considered when interpreting the results. Firstly, Given the relatively small size of our dataset, we initially relied on handcrafted statistical features (such as mean, kurtosis, derivatives, etc.) extracted from AU sequences, primarily due to the interpretability offered by traditional models. This approach allowed us to gain valuable insights into the role of specific features in pain expression and aided in identifying the most relevant AUs for further analysis. We then transitioned to deep learning models (RNN, LSTM, TCN, and Transformer) to explore the potential of end-to-end learning using raw AU sequences. However, their performance may be limited by the smaller dataset. Future work will benefit from larger datasets, enabling the more direct application of end-to-end deep learning models, which would better capture the complex temporal patterns in pain recognition.

We performed the partitioning and external validation at a single center. The single-center design and the relatively homogeneous patient population limit the generalizability of our findings. The sample was drawn from the Beijing Jishuitan Hospital, and the population was composed of a relatively uniform group, which may not fully represent the diversity of the broader patient population. Future research could address this limitation by validating the model on external, multicenter datasets to enhance its robustness and generalizability across different cohorts.In addition, our data collection was conducted in a controlled environment, accounting for factors such as lighting and patient posture. A clinical monitoring system would need to comprehensively account for these confounding factors.

This study did not specifically address cultural, ethnic, or gender differences in facial pain expression. We acknowledge that these factors may limit the model’s generalizability, as variations in pain expression across cultures, ethnicities, and genders could affect its performance in diverse populations. Future research should incorporate these variables to enhance the model’s applicability across broader demographics. Although several relevant studies on pain recognition in ICU settings exist, most of these studies rely on publicly available datasets for validation and do not provide open-source code, making direct comparison with existing work unfeasible. However, we hope this work highlights the gap that exists when systems trained solely on healthy subjects are directly applied in clinical settings.

## Conclusion

This study collected facial data from ICU patients to investigate the expression of facial AUs in response to pain in clinical environments. Transitioning from laboratory settings to clinical applications, this research introduces a reliable temporal machine learning framework for the automatic prediction of patients’ pain states. The method, which does not rely on prior statistical features, autonomously extracts facial pain characteristics, significantly enhancing both predictive accuracy and model robustness. This approach provides scientific evidence for personalized pain management and clinical decision-making.

## Data Availability

The datasets generated and/or analysed during the current study are not publicly available due to patient privacy concerns but are available from the corresponding author on reasonable request.
